# The Role and Mechanism of Vascular Aging in Geriatric Vascular Diseases

**DOI:** 10.14336/AD.2024.0717

**Published:** 2025-08-01

**Authors:** Shouyao Zhang, Bo Xia, Bill Kalionis, Huan Li, Xinyan Zhang, Xinghe Zhang, Shijin Xia

**Affiliations:** ^1^The Second Clinical Medical College, The Second Affiliated Hospital, Yunnan University of Chinese Medicine, Kunming, China.; ^2^Master of Science in Computer Science, Sofia University, Palo Alto, CA, USA.; ^3^Department of Maternal-Fetal Medicine Pregnancy Research Centre, Royal Women’s Hospital, Parkville, Australia.; ^4^Department of Medical Information Service, The General Hospital of Western Theater Command, Chengdu, China.; ^5^Shanghai Institute of Geriatrics, Huadong Hospital, Fudan University, Shanghai, China.

**Keywords:** Vascular aging, geriatric diseases, inflammaging, vascular endothelial cells, oxidative stress, vascular wall calcification, telomere dysfunction

## Abstract

Vascular aging is the pathological basis for the aging of various organ systems in the human body and is a common pathogenesis leading to the development of atherosclerosis, Alzheimer's disease, and other conditions among older adults. Aging is characterized by accelerated pulse wave velocity, thickening of the carotid artery intima-media, and decreased vascular dilation function. Signaling pathways such as mTOR, AMPK, NF-κB, Klotho, SIRT, and other key proteins are likely involved in these processes. The detection of biomarkers related to vascular aging, including senescence-associated β-galactosidase, endothelial progenitor cells, circulating endothelial microparticles, and exosomal miRNAs, aids in assessing vascular status and prognosis. Repairing endothelial injury, reducing oxidative stress-inflammatory responses, and restoring mitochondrial and telomere functions are reliable measures to counter vascular aging. In summary, research on vascular aging is the driving force that will provide rational strategies to intervene in geriatric vascular diseases and achieve the long-term goal of healthy aging.

The pathological basis of various cardiovascular and cerebrovascular diseases is vascular aging. The incidence of diseases related to vascular aging, such as atherosclerosis, hypertension, and diabetes, is rising as the global aging population increases. The total annual expenditure on diabetes in the United States is estimated to be 412.9 billion dollars, and the average annual medical expenditure of diabetic patients is 19,736 dollars. Due to the decrease of employment rate and attendance caused by disability, the indirect productivity loss is as high as 64.1 billion dollars [1]. In developing countries, the situation is also different. In China, 330 million people suffer from cardiovascular diseases, and two out of every five deaths are due to cardiovascular diseases. It is the main reason for the increase of morbidity and mortality [2]. Estimates are that by 2030, there will be 27 million people suffering from hypertension, 8 million people suffering from coronary heart disease and 4 million people suffering from stroke [3, 4]. Clearly, this will impose a heavy financial burden on already strained healthcare systems, further increase healthcare costs and reduce productivity. Moreover, an increasingly unhealthy aging population will be a burden on the whole of society and particularly on families and caregivers. Therefore, in-depth research is very important to determine the characteristics and mechanisms of vascular aging in order to prevent and treat vascular diseases in older adults and provide reasonable strategies to delay aging.

## 1. The concept of vascular aging

**Figure 1.**
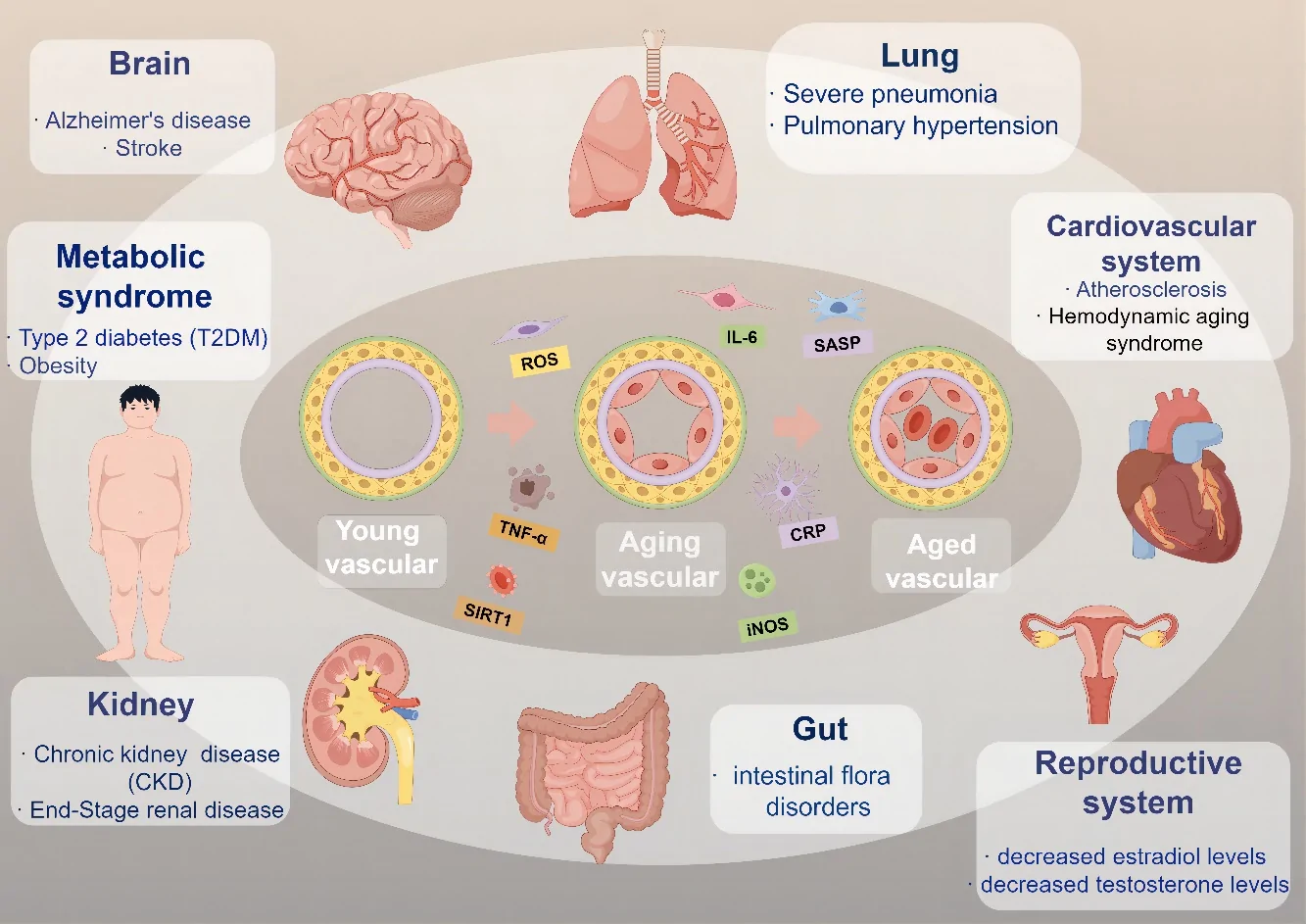
**Changes in human organs related to vascular aging**. Vascular aging is closely correlated with diseases such as Alzheimer's, chronic kidney disease, and atherosclerosis and affecting multiple organs. (Ilustrated using Figdraw. Figdraw.com).

Vascular aging refers to the structural abnormalities and functional degenerative changes that occur in blood vessels with age. According to the Chinese Expert Consensus on Clinical Assessment and Intervention of Vascular Aging [5] and the expert consensus issued by the Aging Biomarker Consortium (ABC) in 2023 [6], the functional manifestations of arterial aging include increased vascular stiffness, altered sensitivity to vascular active factors, decreased angiogenesis capacity, and increased secretion of inflammatory factors. At the morphological level, vascular aging is manifested by increased intima-media thickness, disordered elastic fibers, increased collagen deposition, and disordered arrangement of vascular smooth muscle cells. At the cellular level, morphological abnormalities in endothelial cells and smooth muscle cells are present such as flattened endothelial cells and hypertrophic smooth muscle cells. Further features are increased collagen and matrix mucopolysaccharide deposition and decreased elastic fibers. The aging of vascular morphology and function forms the basis for various vascular diseases and indeed aging of the entire body [7] (Fig. 1).

## 2. Characteristics of vascular aging

### 2.1. Pulse wave velocity is accelerated

Pulse wave velocity (PWV) is the most commonly used method for detecting vascular stiffness. Clinically, carotid-femoral PWV (cfPWV), brachial-ankle PWV (baPWV) are commonly used to represent the conduction velocity of arterial pulse waves formed when the heart pumps blood along the arterial wall from the proximal to the distal end. The ankle-brachial index (ABI) can indicate the blockage of large and medium-sized arteries between the ankle and brachial arteries. Thus, a combination of baPWV and ABI is often used to assess vascular stiffness and has a high predictive value for cardiovascular disease diagnosis. Between the ages of 20 and 70, studies show that baPWV increases from approximately 1200 cm/s to 1500 cm/s, an increase of about 25%, which demonstrates significant age-related changes [8].

### 2.2 Increase in carotid intima-media thickness (IMT)

The intima media thickness of the carotid artery (IMT) is the distance between the lumen of the carotid artery and the outer membrane interface. Ultrasound measurement of IMT has become an important tool for detecting arterial stiffness. IMT thickening is a landmark structural change of vascular aging, which is closely related to age [9], carotid artery IMT measurement helps to assess the risk and incidence rate of brain and cardiovascular diseases [10]. Research has shown a moderate correlation between IMT and vascular age, which tends to accelerate in women in their 60s and men in their 70s, indicating that IMT is an effective tool for clinical assessment of vascular aging [11].

### 2.3 Impaired arterial flow-mediated dilation (FMD)

Flow-mediated dilation (FMD) is an important indicator for evaluating endothelial vasodilation function. Endothelial cells are the first natural barrier of blood vessels and play an important role in the aging process of blood vessels. Endothelial cell aging is associated with a decrease in nitric oxide (NO) release, resulting in impaired endothelial vasodilatation function and weakened vascular relaxation ability [12]. The principle of FMD detection is to measure using ultrasound, the temporary change in the diameter of the brachial artery under shear stress. The higher the value of vascular relaxation function, the better the endothelial function of the subject; conversely, this indicates impaired endothelial function [13]. Studies report patients with chronic kidney disease and metabolic syndrome have varying degrees of impairment in their FMD index, which increases the risk of cardiovascular events [14]. FMD is also an independent predictor for the severity and prognosis of patients with pulmonary hypertension and chronic obstructive pulmonary disease [15].

### 2.4 Biomarkers of vascular aging

Senescence-associated β-galactosidase (SA-βGal) is widely recognized as one of the biomarkers of endothelial cell senescence. SA-βGal increases significantly in senescent vascular smooth muscle cells, and its expression level positively correlates with the degree of arterial calcification [16]. Endothelial progenitor cells (EPCs) are endothelial precursor cells derived from the bone marrow and play a crucial role in maintaining vascular endothelial function. With aging, a decrease in circulating EPCs may cause endothelial dysfunction and impaired arterial elasticity [17]. Therefore, changes in EPC levels serve as a biomarker of vascular aging. Endothelial microparticles (EMPs) are micro-particles shed from damaged or apoptotic endothelial cell membranes. An increase in EMPs is considered an important marker of endothelial damage and increased vascular stiffness [18]. The integrity of vascular endothelial cell function is also regulated by vascular endothelial growth factor (VEGF) [19]. Mice treated with VEGF show a longer lifespan, indicating that VEGF is a potential target for regulating the process of vascular aging [20]. Fibulin-1 is a vital extracellular matrix protein of vascular smooth muscle cells, is overexpressed in people with atherosclerosis and positively correlates with increased baPWV, making it a potential marker of arterial stiffness [21]. In recent years, studies show that exosomal miRNAs may be involved in angiogenesis, repairing vascular damage by regulating the microenvironment, endothelial function and thereby controlling vascular aging [22]. For example, exosomal miR-675 from stem cells regulates endothelial dysfunction in senescent blood vessels by inhibiting the transforming growth factor-β1 (TGF-β1)/p21 pathway [23]. Therefore, exosomes are also considered potential regulators of vascular aging.

## 3. Diseases related to vascular aging

### 3.1 Geriatric lung diseases

Pulmonary hypertension (PH) is a common pulmonary vascular disease characterized by elevated pulmonary artery pressure combined with varying degrees of right heart failure. Epidemiological studies show the prevalence of PH in the general population is about 1%, but increases to 10% in people over 60 years old, suggesting that age may be an independent risk factor for the development of PH [24]. With aging, the arterial walls thicken, and their elasticity decreases. Hardened vessel walls reduce arterial compliance, elevating systolic blood pressure and ultimately affecting cardiac and pulmonary function, known as hemodynamic aging syndrome [25, 26]. Studies report that p21 and p16 are associated with aging-related vascular remodeling in PH. Compared to controls, the mRNA and protein levels of p21 are significantly elevated in pulmonary artery smooth muscle cells of aged mice [27, 28]. Chronic obstructive pulmonary disease (COPD) is another common geriatric lung disease. Noureddine et al. measured telomere length and senescence-associated molecules such as p16 and p21 in pulmonary vascular smooth muscle cells from 124 COPD patients. They found that senescence is a crucial factor contributing to vascular remodeling [29]. Vascular inflammatory senescence is widely involved in the pathogenesis of geriatric lung diseases. Proinflammatory cytokines such as IL-1β, IL-6, TNF-α, and CRP are significantly elevated in older adults with severe pneumonia, in patients with pulmonary hypertension, and in patients with other conditions [30-32]. These cytokines also act on senescent cells, accelerating their aging process [33]. The decline in cardiopulmonary function caused by vascular aging is unavoidable. Active symptomatic treatment and healthy lifestyle habits help reduce the risk of atherosclerosis and cardiovascular diseases.

### 3.2 Cerebrovascular diseases

The brain is an organ that heavily relies on blood supply for its structural and functional integrity. Contributing to this are dense blood vessels with low resistance in small vessels, fast blood flow, and blood flow accounting for about 20% of cardiac output. With vascular aging, vascular stiffness gradually increases, and PWV accelerates, making it prone to pulsatile microvascular damage. This may lead to ischemia or hemorrhage in tiny brain vessels, a common age-related change in various cerebrovascular diseases. A carotid-femoral pulse wave velocity ≥6.66m/s is an independent predictor of poor prognosis after acute stroke [34, 35]. Arterial stiffness is also an independent determinant of Alzheimer's disease (AD). Studies report that individuals with higher arterial stiffness have lower integrity of gray and white matter in the brain [36], and have decreased self-regulation ability of cerebral blood vessels, which lead to reduced cerebral blood flow. These features significantly impact the brain's cognitive function [37]. Another major age-related change in AD is the deposition of amyloid β-protein (Aβ). Aβ deposits in the walls of cerebral arteries and capillaries, causing so-called cerebral amyloid angiopathy (CAA). CAA occurs in 85% to 95% of AD patients, resulting in vascular cell apoptosis, reduced vascular wall strength, decreased cerebrovascular self-regulation, and impaired blood-brain barrier integrity. This leads to cerebral microinfarcts and microbleeds, which further exacerbates cognitive impairment [38].

### 3.3 Atherosclerosis

Atherosclerosis is one of the key characteristics of vascular aging and a leading cause of death and disability in older adults worldwide [39]. Age is a significant risk factor for the development of atherosclerosis, characterized by endothelial cell dysfunction, β-amyloid clearance disorders, oxidative stress-inflammatory responses, and telomere dysfunction [40, 41]. Increasing evidence suggests a biological correlation between cellular senescence and atherosclerosis. The occurrence of atherosclerosis in older adults is closely related to the regulation of endothelin-1(ET-1), nitric oxide (NO) and angiotensin II levels by vascular endothelial cells [42]. Vascular smooth muscle cells (VSMC) play a key role in the formation of atherosclerosis. VSMC with autophagy defect will lead to calcification of arterial wall and promote age-related changes in vascular system [43]. In addition, amyloid proteins such as apolipoprotein A-1 (Apo1) deposit within the aortic wall, leading to vascular amyloidosis [44], these factors ultimately contribute to the development of atherosclerosis. Aged blood vessels promote the formation of atherosclerotic plaques, which in turn exacerbate vascular aging, creating a vicious cycle. Active anti-inflammatory and lipid-lowering therapies, along with lifestyle modifications remain recommended interventions.

### 3.4 Metabolic syndrome

The increase in the prevalence of metabolic syndrome is closely related to vascular aging. The accumulation of advanced glycation and products (AGEs) in tissues is a characteristic feature of vascular aging [45]. The enhancement of AGEs in diabetic patients interferes with the homeostasis of the blood vessel wall, triggering excessive production of ROS and the release of proinflammatory cytokines such as IL-6 and IL-1β [46], which leads to decreased numbers of pancreatic beta cells and dysfunction [47]. These are all processes involved in the pathogenesis of Type 2 Diabetes Mellitus (T2DM). Another manifestation of metabolic syndrome is obesity. Fasting and postprandial blood flow to adipose tissue may both be impaired, leading to altered lipid metabolism and an increased risk of cardiovascular disease [48]. Additionally, obesity-induced gut microbiota dysbiosis cause oxidative stress and vascular dysfunction [49]. Science-based nutrition or a healthy diet, exercise, medication, and other means can change the composition of the microbiota and thereby inhibit inflammation and age-related vascular dysfunction in obese patients.

## 4. Mechanisms of vascular aging

### 4.1 Changes in vascular structure

#### 4.1.1 Vascular wall calcification

Vascular wall calcification refers to the abnormal deposition of calcium and phosphorus in the blood vessel wall, a biological process similar to bone formation [50]. Recent studies report the occurrence of vascular calcification is closely related to aging and is the pathological basis of many common diseases in older adults. For example, diabetic patients often have extensive medial calcification of the lower extremity peripheral arteries [51]. For patients with chronic kidney disease, the risk of coronary vascular calcification increases by 6.7% for each additional year of age, and almost all patients with end-stage chronic kidney disease have calcium and phosphorus metabolism disorders in the vascular wall [52]. In addition, multiple cytokines and growth factors are involved in the process of vascular calcification. Among them, proinflammatory cytokines (IL-6, TNF-α) [53], osteoprotegerin [54], sclerostin [55], matrix Gla protein (MGP), and fibroblast growth factor (FGF-23) and its receptor Klotho protein [56, 57] promote/antagonize vascular calcification by regulating inflammation, oxidative stress, and osteogenesis-related signaling pathways. Vascular calcification is a very common pathological phenomenon in the older adult population, and the development of effective prevention and treatment methods is of great significance for controlling the prevalence and mortality of cardiovascular and cerebrovascular diseases in older adults.

#### 4.1.2 Microvascular damage

**Figure 2.**
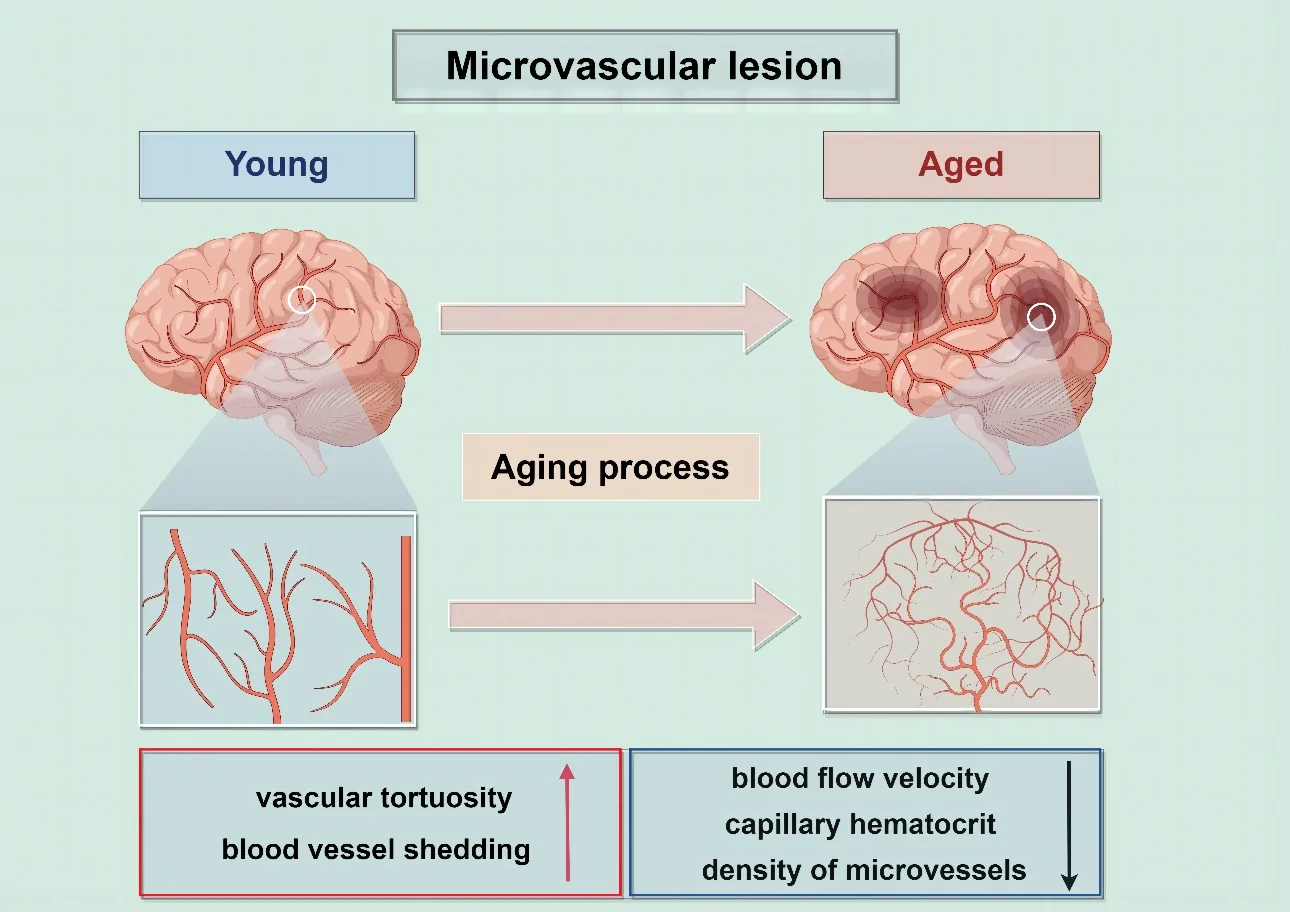
**Age-related microvascular damage**. Aging induces tortuosity and sparseness of cerebral microvascular, which further leads to reduced blood flow velocity and blood vessel shedding. (Illustrated uisng Figdraw, Figdraw.com).

Cerebral microvessels generally refer to the microvascular network between small arteries and veins, with an average diameter of 7-9μm. They provide nutrients, transport metabolites, and maintain the blood-brain barrier. During aging, the morphology and function of cerebral microvessels undergo varying degrees of abnormal changes (Fig. 2). Compared to young rodents, aged rodents had reduced cerebral microvascular blood flow velocity, increased vascular tortuosity and significantly reduced hematocrit [58, 59]. Another study using magnetic resonance angiography involving 100 subjects reported that with increasing age, there was a trend of vascular shedding in the left and right middle cerebral arteries, as well as in the anterior and posterior cerebral artery regions of the subjects [60]. After the age of 60, the total number of circulating blood vessels was significantly reduced [61], and in the gray matter of the brains of AD patients, blood flow decreased by about 42% [62]. The above evidence suggests that microvascular damage is a common pathological feature of vascular aging in older adults, and its harm should not be underestimated.

### 4.2 Changes in vascular microenvironment

#### 4.2.1 Oxidative stress

Oxidative stress is a cellular physiological process characterized by an imbalance between ROS and antioxidants, leading to oxidative damage in cells and consequently causes chronic systemic inflammation [63]. Inflammation is recognized as an endogenous factor of aging. Older adults may experience aseptic, low-grade chronic inflammation, resulting in an imbalance of inflammatory homeostasis. This condition, which intensifies with age, is called inflamm-aging and it contributes to the occurrence and progression of various age-related diseases. Reducing inflammation may help delay the aging process [64]. The term inflamm-aging was first proposed by Franceschi et al. in 2000 [65]. Since then, inflamm-aging has been intensively studied to determine its molecular mechanisms in age-related diseases [66].

The intimate relationship between oxidative stress, inflammation, and aging, led some scholars to propose the theory of oxidative-inflammatory-aging (Oxi-Inflamm-Aging) [67, 68]. According to this theory, oxidative stress induces vascular endothelial and smooth muscle cells to produce proinflammatory factors such as IL-6, TNFα, and nitric oxide synthase (NOS), disrupting the metabolic microenvironment of vascular cells. Consequently, there is increasing cell apoptosis, which ultimately promotes aging [69, 70], especially in a hypoxic environment. ROS accumulates in large quantities, and the senescence-associated secretory phenotype (SASP) increases, exacerbating the aging process [71]. Additionally, telomere shortening, and DNA double-strand breaks induced by oxidative stress are considered important factors in endothelial cell senescence [72]. This suggests that oxidative stress promotes aging through multiple pathways. While natural aging cannot be avoided, reducing calorie intake, increasing exercise, and supplementing antioxidants such as Vitamin E and Vitamin A may help reduce the damage caused by oxidative stress to human blood vessels.

#### 4.2.2 Telomere dysfunction

Telomeres are protective structures at the ends of chromosomes that play a crucial role in maintaining continuous cell division and slowing down aging [73]. Telomeres shorten with each cell division, making the maintenance of telomere length particularly important. Numerous studies confirm telomere length is closely related to vascular aging. The T-loop formed at the end of telomeres helps prevent chromosome double-strand breaks, and the destruction of the T-loop structure is called "uncapping." Telomere uncapping is another physiological process associated with vascular aging. Morgan et al. [74] found that reducing the expression of telomeric repeat-binding factor 2 (TRF2) leads to telomere uncapping, which reduces vascular endothelial dilatation capacity, increases proinflammatory signaling and oxidative stress, and induces vascular aging [75]. Therefore, telomere shortening is considered an important marker for measuring vascular aging.

#### 4.2.3 Reduced autophagy activity

Autophagy is the main degradation process in eukaryotic cells, driven by an evolutionarily conserved mechanism involving autophagy-related proteins (ATG). Studies reveal that overexpressing ATG5 reduces the proliferation of melanoma cells and delays aging progression. In contrast, knocking out the autophagy-related gene ATG6 significantly shortens the lifespan of DAF-2 mutants [76, 77], which may be related to increased levels of oxidative stress and inflammation, reduced bioavailability of nitric oxide, and impaired vasodilatation function [78, 79]. Acetylation modification has an important influence on autophagy regulation. SIRT1 is a key target of acetylation modification. Evidence suggests that increasing SIRT1 activity in senescent human umbilical vein endothelial cells reduces the acetylation of p53 and partially reverses vascular aging [80]. In contrast, inhibiting SIRT1 activity in Wistar rat aortas leads to increased ROS in the vessel wall, inducing vascular aging [81]. These findings suggest autophagy plays a crucial role in maintaining homeostasis within the body. However, it should be noted that autophagy has a dual nature, and excessive activation of autophagy may promote tumor cell growth to some extent [82], Therefore, the complex relationship between autophagy and aging requires further exploration.

#### 4.2.4 Mitochondrial dysfunction

**Figure 3.**
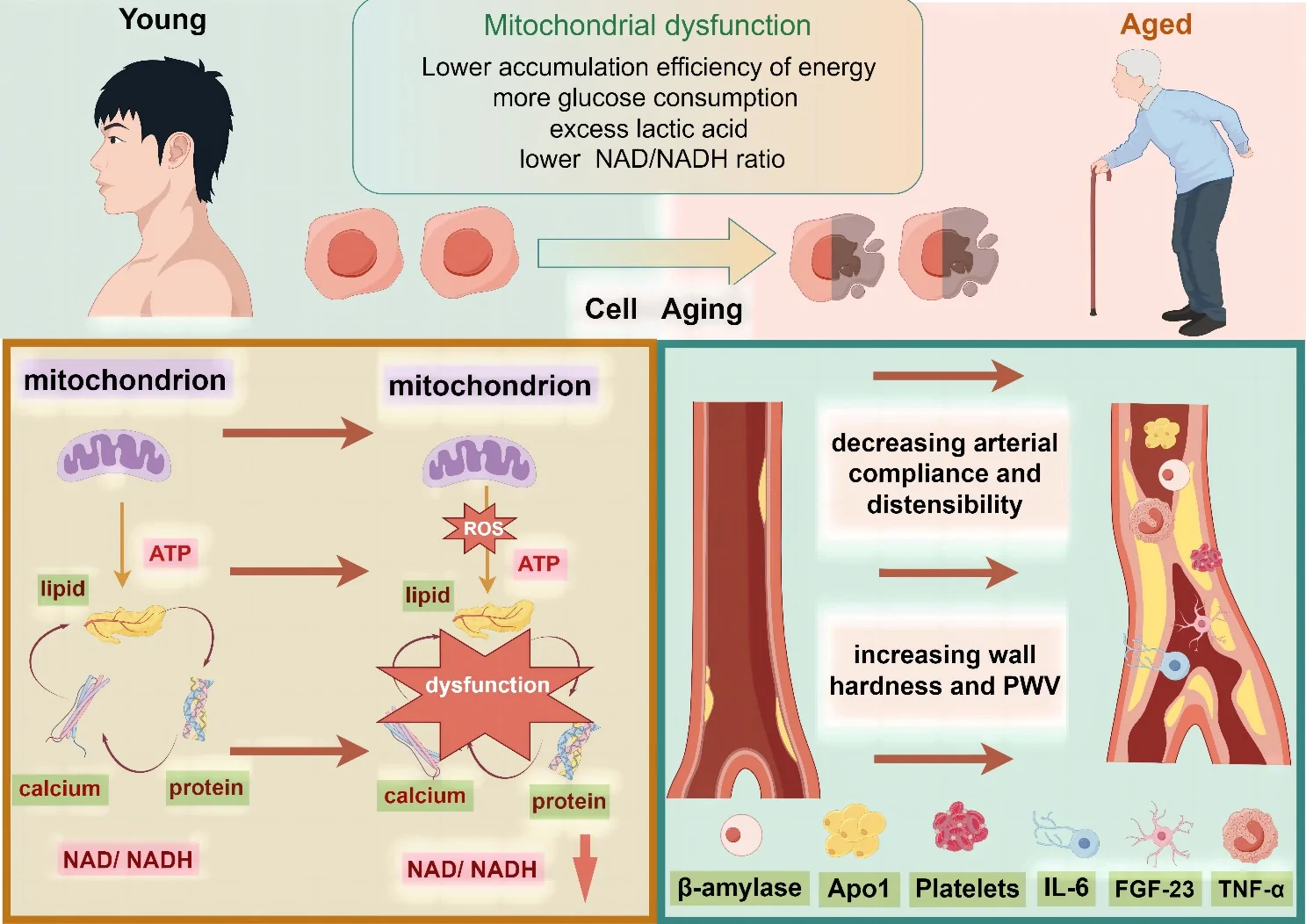
**Age-related changes in the mitochondrion and blood vessel.** (**A**) Aging leads to mitochondrial dysfunction, gradually reducing the efficiency of energy metabolism; (B) Aging increases vascular hardness and PWV, while decreasing compliance and distensibility. (Illustrated by Figdraw, Figdraw.com).

Mitochondria are key organelles that regulate cellular metabolism, ATP production, ROS generation, and cell death. Mitochondrial dysfunction is a hallmark of aging [83]. Cellular energy mainly comes from the oxidative decomposition of organic substances such as sugars, lipids, and proteins. Mitochondria play a crucial role in this process by producing adenosine triphosphate (ATP) to provide energy for biochemical reactions (Fig. 3A). Dysfunctional mitochondria reduce the accumulation efficiency of calcium, lipids, and proteins, increase glucose consumption and produce excess lactic acid, and decrease the NAD/NADH ratio. This ultimately leads to cellular senescence, by the process referred to as mitochondrial dysfunction-associated senescence [84] (Fig. 3B). As the central organelle of the cellular functional network, mitochondria exchange substances with surrounding organelles. Current research suggests that mitochondria and the endoplasmic reticulum collaborate to regulate processes such as calcium signaling, ROS signaling, lipid metabolism, autophagy, and apoptosis. This collaboration plays a significant role in the development of aging-related cardiovascular diseases [85]. These findings suggest that impaired mitochondrial communication processes contribute to the senescence of healthy cells.

#### 4.2.5 Decline in sex hormone levels

Sex hormones decline with age, which to some extent accelerates vascular aging. A meta-analysis reported that compared to the control group, patients with cardiovascular diseases have significantly lower testosterone levels, and are more likely to exhibit atherosclerotic plaques and higher levels of C-reactive protein (CRP) [86]. Some evidence suggests that the decline in estradiol levels during perimenopause in women may exacerbate endothelial dysfunction to some extent. This implies that estrogen provides endothelial protection for women, which disappears as they enter perimenopause [87]. Similarly, compared to men with higher testosterone levels, those men with lower testosterone levels are more prone to endothelial dysfunction. However, testosterone therapy does improve endothelial function after 6 and 12 months of treatment [88].

#### 4.2.6 Platelet aggregation

Platelet aggregation is closely related to vascular aging. As people age, senescent platelets adhere and aggregate more readily, interacting with immune cells to produce inflammatory cytokines, promoting thrombosis, and thus increasing the risk of cardiovascular diseases [89, 90]. The COMPASS trial shows that antiplatelet and anticoagulation therapies help to improve the final outcome of coronary artery and cerebrovascular disease [91]. This suggests that platelet aggregation is associated with cardiovascular adverse events in older adults. In addition, ROS play a key role in the platelet activation mechanism during the aging process, and increased oxidative stress may lead to accelerated aging. Therefore, platelet oxidative stress may serve as a target for cardiovascular risk in older adults [92]. As a simple and effective blood indicator, platelet aggregation may provide a reference for the diagnosis and treatment of older adult associated vascular aging-related diseases.

## 5 Intervention strategies based on vascular aging mechanisms

### 5.1 Improving vascular endothelial damage

Studies confirm that drugs, such as rapamycin, regulate SKN-1 and DAF-16, improve vascular endothelial damage and improve blood pressure and carotid IMT [93], providing evidence of their vasculoprotective properties. The renin-angiotensin system plays a role in controlling vascular aging by reducing Sirt3 expression through Ang II signal transduction [94]. The protective role of atorvastatin in the cardiovascular system is widely recognized. By upregulating the ubiquitin ligase WWP2, atorvastatin offsets angiotensin II-induced vascular endothelial damage and improves vascular aging in hypertensive mice [95]. Diabetes is an important independent factor contributing to vascular aging. The first-line drug metformin significantly improves the survival of subjects by initiating the AMP-activated protein kinase (AMPK) and inhibiting the mTOR pathway to regulate biological aging mechanisms [34, 96].

Moderate exercise helps control arteriosclerosis. A Spanish study reported a positive correlation between vascular aging and sedentary time, and a negative correlation with physical activity. The carotid-femoral pulse wave velocity (cfPWV) was 26% higher in sedentary individuals and they showed a more pronounced age-related decline in endothelial function when compared with age-matched athletes [97]. Notably, previously sedentary, healthy middle-aged and older men, who underwent 8-12 weeks of regular, moderate intensity aerobic exercises showed improved forearm capillary and brachial artery endothelial function close to levels detected in healthy, young men [98]. This indicates that moderate exercise is beneficial for maintaining vascular endothelial health.

### 5.2 Reducing oxidative stress and inflammation

Oxidative stress triggers vascular inflammation, promotes telomere shortening and DNA double-strand breaks [72]. Reducing oxidative-inflammatory responses is an important strategy for intervening in vascular aging. Senolytics therapy, which selectively eliminates senescent cells, shows potential in extending biological lifespan. A mixture of dasatinib, a kinase inhibitor, and quercetin, a flavonol, improves vascular endothelial function and reduces chronic inflammation and its harmful effects [99]. Key proteins in many inflammatory and oxidative stress signaling pathways (i.e. mTOR, AMPK, SIRT, and Klotho) reduce oxidative stress, decrease inflammatory responses, and improve vascular function [100-103]. A newly developed therapy employs the stilbenoid resveratrol, which exerts anti-inflammatory effects by inhibiting NF-κB and JAK/STAT signaling pathways, helping to slow the progression of vascular aging [104].

Additionally, a calorie-controlled diet is a beneficial approach to improve the vascular inflammatory microenvironment and prevent vascular aging. Adhering to a high-fiber breakfast (including oat bran porridge with low-fat milk, whole wheat bread, fruits, etc.) over the long term significantly improves sagittal abdominal diameter, and reduces inflammatory markers CRP and TNF-R2 [105], Moreover, adjusting dietary habits significantly alter the degree of calcification in the abdominal aorta and help reduce the risk of death [106]. This may be related to reduced oxidative damage, activated transcription factors, and control of endoplasmic reticulum stress responses [107].

### 5.3 Restoring mitochondrial quality control

Restoring mitochondrial quality control may provide a protective strategy against vascular aging. Studies report that oral administration of spermidine enhances mitochondrial autophagy in older adult hyperlipidemic mice, which prevents an increase in aortic IL-6 and Parkin protein, reduces mitochondrial dysfunction, and decreases the incidence of atherosclerosis [108]. Carbonyl cyanide p-(tri-fluromethoxy) phenyl-hydrazone(FCCP), is an effective oxidative phosphorylation uncoupler that enhances mitochondrial autophagy [109], and reduces age-related elevations of Parkin, Toll-like receptor 9 (TLR9), and myeloid differentiation primary response 88 (MyD88) in older adult’s aorta. Furthermore, both calorie restriction and aerobic exercise are reported to delay vascular aging. Aerobic exercise promotes mitochondrial biosynthesis and mitochondrial oxidative capacity, inhibits mtDNA mutations, and restores respiratory chain assembly as well as mitochondrial morphology and function. These changes attenuate pathological changes in aging blood vessels [110].

### 5.4 Targeting markers of telomere damage

Telomere dysfunction potentially results in age-related abnormal vascular functioning. Deletion of the telomere repeat-binding factor 2 (TRF2) component of shelterin, which is a telomere-binding protein complex, resulted in telomere dysfunction and induced endothelial dysfunction in large and small arteries [111]. This suggests that a dysfunctional shelterin complex may cause age-related telomere uncapping. Manipulating this complex may improve telomere dysfunction. Emerging evidence shows that a long non-coding RNA transcribed at telomeres, called TERRA (Telomeric Repeat-containing RNA), which regulates telomere maintenance and chromosome capping mechanisms. TERRA interacts with the shelterin complex to maintain telomere structural stability [112], and thereby shows potential as a novel therapeutic target.

### 5.5 Improving sex hormone levels

Testosterone therapy induces serum testosterone and Gas6 levels, improving cellular senescence and vascular remodeling in aging mice, thereby exerting a protective effect on vascular aging [113]. In postmenopausal women, estradiol therapy has improved endothelial function. Transdermal treatment with 17β-estradiol and norethindrone acetate enhances arterial compliance [114]. Importantly, estradiol and exercise may act synergistically to increase resistance to oxidative damage by activating and phosphorylating eNOS to release NO [115]. Improving sex hormone levels may serve as a therapeutic strategy to ameliorate vascular aging.

## 6. Conclusions

Cardiovascular diseases related to vascular aging are the main causes of disability and death in old adults globally [116]. This study summarizes the current knowledge of the molecular biological mechanisms of vascular aging and finds that mTOR, AMPK, NF-κB, Klotho and SIRT signaling pathways and other key proteins, are potentially important in the process of vascular aging. Measuring β-galactosidase, exocrine miRNA and other vascular aging biomarkers is helpful for evaluating vascular status. Reliable clinical measures to deal with vascular aging include actively controlling blood pressure, blood sugar and blood lipids, reducing oxidative stress-inflammatory reactions, restoring mitochondrial and telomere functions, and reducing platelet aggregation. In the future, emerging technologies such as machine learning algorithms will improve the success and efficiency of clinical trials by detecting clusters of signals and symptoms to identify adverse events and predict possible safety issues. Artificial intelligence will help molecular biology research with the use of “deep learning” algorithms to identify patterns and connections from “omics” bioinformatics analyses, which will allow the creation of predictive tools based on patterns that will aid in the diagnosis of vascular diseases in older adults and the development of novel treatment strategies [117, 118].

Although arterial stiffness develops with age, there are still studies showing that adolescents may develop arterial stiffness prematurely [119], and the occurrence of arteriosclerosis also shows significant racial and gender differences. The growth rate of arterial stiffness of African Americans is significantly higher than that of European Americans, and that of men is higher than that of women [120]. An unhealthy lifestyle and social factors will also affect vascular health. High cholesterol level, smoking, diabetes and obesity are unfavorable factors for arteriosclerosis, while a good living environment (such as air quality) [121], reducing sedentary time and moderate exercise will help prevent or delay the occurrence and development of arteriosclerosis.

There are limitations to this review, for example, the sample size of some cited documents is small, and there may be potential bias or inconsistency in methods. Based on large-sample, multi-center and high-quality clinical randomized controlled research, the trend of future research is to produce a unified clinical diagnosis and treatment guide and explore the methods of early identification and intervention of vascular aging.
